# Population-Based Study on Risk Factors for Tumor-Positive Resection Margins in Patients with Gastric Cancer

**DOI:** 10.1245/s10434-019-07381-0

**Published:** 2019-04-22

**Authors:** Leonie R. van der Werf, Charlotte Cords, Ivo Arntz, Eric J. T. Belt, Ivan M. Cherepanin, Peter-Paul L. O. Coene, Erwin van der Harst, Joos Heisterkamp, Barbara S. Langenhoff, Bas Lamme, Mark I. van Berge Henegouwen, Sjoerd M. Lagarde, Bas P. L. Wijnhoven

**Affiliations:** 1 Department of Surgery, Erasmus University Medical Centre Rotterdam, Rotterdam, The Netherlands; 2 Department of Surgery, Bravis Hospital, Roosendaal, The Netherlands; 3 Department of Surgery, Albert Schweitzer Hospital, Dordrecht, The Netherlands; 4 Department of Surgery, Maasstad Hospital, Rotterdam, The Netherlands; 5 Department of Surgery, Elisabeth Tweesteden Hospital, Tilburg, The Netherlands; 6 Department of Surgery, Cancer Center Amsterdam, Academic Medical Centre, University of Amsterdam, Amsterdam, The Netherlands

## Abstract

**Background:**

Radical gastrectomy is the cornerstone of the treatment of locally advanced gastric cancer. This study was designed to evaluate factors associated with a tumor-positive resection margin after gastrectomy and to evaluate the influence of hospital volume.

**Methods:**

In this Dutch cohort study, patients with junctional or gastric cancer who underwent curative gastrectomy between 2011 and 2017 were included. The primary outcome was incomplete tumor removal after the operation defined as the microscopic presence of tumor cells at the resection margin. The association of patient and disease characteristics with incomplete tumor removal was tested with multivariable regression analysis. The association of annual hospital volume with incomplete tumor removal was tested and adjusted for the patient- and disease characteristics.

**Results:**

In total, 2799 patients were included. Incomplete tumor removal was seen in 265 (9.5%) patients. Factors associated with incomplete tumor removal were: tumor located in the entire stomach (odds ratio (OR) [95% confidence interval (CI): 3.38 [1.91–5.96] reference: gastroesophageal junction), cT3, cT4, cTx (1.75 [1.20–2.56], 2.63 [1.47–4.70], 1.60 [1.03–2.48], reference: cT0-2), pN+ (2.73 [1.96–3.80], reference: pN−), and diffuse and unknown histological subtype (3.15 [2.14–4.46] and 2.05 [1.34–3.13], reference: intestinal). Unknown differentiation grade was associated with complete tumor removal (0.50 [0.30–0.83], reference: poor/undifferentiated). Compared with a hospital volume of < 20 resections/year, 20–39, and > 39 resections were associated with lower probability for incomplete tumor removal (OR 0.56 [0.42–0.76] and 0.34 [0.18–0.64]).

**Conclusions:**

Tumor location, cT, pN, histological subtype, and tumor differentiation are associated with incomplete tumor removal. The association of incomplete tumor removal with an annual hospital volume of < 20 resections may underline the need for further centralization of gastric cancer care in the Netherlands.

**Electronic supplementary material:**

The online version of this article (10.1245/s10434-019-07381-0) contains supplementary material, which is available to authorized users.

A radical gastrectomy is one of the most important predictors of survival in patients with gastric cancer.^1^ An nonradical resection, i.e., gastrectomy with a tumor-positive resection margin (incomplete tumor removal), is seen in approximately 1.8–8.4% of patients.^2^ In the Netherlands, the percentage of incomplete tumor removal is used as one of the quality indicators of gastrectomies. Between 2011 and 2016, of all gastrectomies for gastric cancer with curative intent, in 9–13% the tumors were incompletely removed.^3^ This number corresponds with other European outcome registries.^4^,^5^ The British National Oesophago-Gastric Cancer Audit (NOGCA) reported that up to 29% of the gastrectomies performed in individual hospitals had tumor-positive margins.^5^ In the Swedish Register for Esophageal and Gastric Cancer (NREV), the percentage of incomplete tumor removal/unknown resection status was 17%.^4^

Gastric cancer surgery might involve tumor-positive margins on the distal side (duodenum), proximal (gastric remnant or esophagus), or circumferential. With the current literature, it is unknown which side is most involved. The Dutch national guideline, nevertheless, recommends a proximal and distal resection margin of 60 mm.^6^

Awareness of increased risk for incomplete tumor removal may prevent this undesirable outcome. However, data on factors associated with incomplete tumor removal, including preoperative risk assessment models, are scarce. So far, retrospective studies have reported on cohorts from single centers, and only few patients were included.^2^,^7^ Also, surgical expertise and quality assurance may play an important role. Because individual surgical volume data are difficult to retrieve, annual hospital volume is a widely accepted proxy for surgical experience. For complex surgery, including upper gastrointestinal surgery, there is evidence that higher hospital volume and individual surgeon volume are associated with improved surgical quality and outcome.^8^–^11^ However, the relation between hospital volume and incomplete tumor removal has never been investigated.

This study was designed to evaluate the factors associated with incomplete tumor removal in a Dutch cohort. Furthermore, we sought the association between hospital volume and incomplete tumor removal.

## Methods

### Study Design

Patient data were retrieved from the Dutch Upper Gastrointestinal Cancer Audit. This surgical audit was initiated in 2011. Hospitals are mandated to register all patients with esophageal or gastric cancer undergoing surgery with curative intent. The DUCA provides insight into the quality of care by reporting validated process and outcome parameters, defined as “quality indicators.”

Because the radicality of an operation is used as one of the quality indicators, the resection status (*R*0, *R*1, *R*2, not applicable, or unknown), as well as the site of the resection margin (proximal, distal, circumferential) in millimeters is registered. For the reporting of the pathological examination of esophageal and gastric cancer, a standardized report is used.^12^ For this study, data on pathology of the resection specimen, patient, tumor, and treatment characteristics were used. Validation of completeness and accuracy of data registration in the DUCA dataset has been performed.^13^ Patient- and hospital identity is anonymous in this database. The study protocol was approved by the DUCA scientific committee.

### Patient Selection

Included were all patients with gastric cancer or cancer of the esophagogastric junction (Siewert type I–III) who underwent gastrectomy between 2011 and 2017 defined as curative by the surgeon at the end of the operation.^14^ Patients were excluded if the resection status or essential elements of the registration were unknown including date of birth, survival status at 30 days after surgery or date of discharge (in case of a hospital stay of > 30 days).

### Outcomes

The primary outcome was complete tumor removal as documented by the pathologist based on examination of the resection specimen. The definition of the College of American Pathologists is used in the DUCA to define the completeness of the tumor removal.^15^ Removal of the tumor is considered complete (*R*0) if no microscopical tumor cells are visible in the margin and incomplete (*R*1 or *R*2) if microscopically or macroscopically tumor cells are visible in the margin. (Patients whom the surgeon defined the resection as complete and curative at the end of the operation, but where the pathological examination showed an *R*2 resection, were included, because this study focuses on the surgeon’s estimation of the resection margins.)

### Statistical Analysis

To compare patient and tumor characteristics between the groups with an *R*0 and *R*1/*R*2 resection, the *χ*^2^ test was used. Univariable and multivariable logistic regression analyses were performed to identify factors associated with incomplete tumor removal. Factors with a *P* value < 0.10 in univariable analyses or with clinical relevance were included in the multivariable analyses. To test whether the explanatory variables are useful in predicting the outcome, the Nagelkerke *R*^2^ and an area under the receiver operating characteristic curve (ROC) was used. By expert opinion, possible factors for the preoperative associated risk model were selected. At the selection of factors for this model, it was decided to choose only patient and tumor characteristics. Treatment characteristics, such as neo-adjuvant chemotherapy and surgical approach, were not selected. Because this could potentially lead to bias, these factors were analysed with descriptive statistics. The factors’ age, Charlson comorbidity score, American Society of Anesthesiologists (ASA) score, tumor location, TNM stage, histological subtype, differentiation grade, and year of surgery were used.^16^ To determine factors that can be used preoperatively to identify patients who are at risk for incomplete tumor removal, the clinical TNM category was preferred for this analysis. However, for N-status of the tumor it was chosen to use pN-stage. The first reason was because an unknown clinical N-stage (cNx) was registered in 13% of patients.^17^ Also, cN-stage and pN-stage do often not correspond, and pN-stage is more reliable.

To test the association of annual hospital volume with the resection status, logistic regression models were used with and without adjustment for case-mix variety. Because centralization has taken place in the Netherlands, analyses were executed in the total cohort of 2011–2017 and stratified for the most recent years 2014–2017. Between 2014 and 2017 hospital volumes were more constant. To address possible confounding caused by differences in treatment strategy between high- and low-volume hospitals, stratified analyses for patients treated with or without neo-adjuvant therapy was performed.

The annual hospital volume in the year of surgery was assigned to each patient. Because the minimum annual hospital volume in the Netherlands is 20 resections per year and to draw clinically relevant conclusions, subsequently, the volume was grouped into three groups: < 20, 20–39, and ≥ 40 resections per year. Missing items were analyzed in a separate group if exceeding 5%.

For all analyses, statistical significance was defined as *P* < 0.05. All analyses were performed with SPSS version 24 (IBM, Armonk, NY).

## Results

**Fig. 1:**
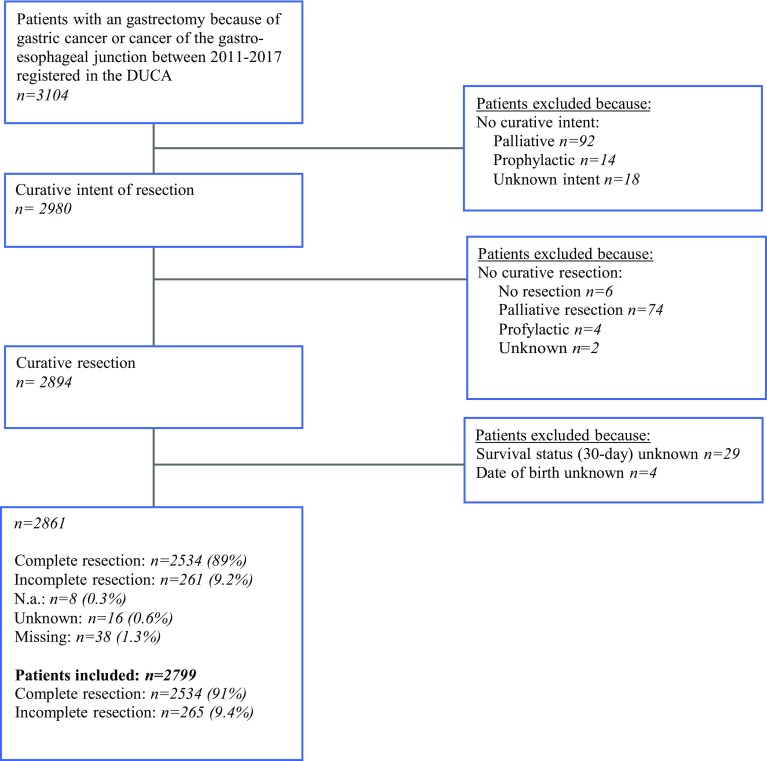
Flowchart inclusion

A total of 2799 patients had undergone a curative gastrectomy according to the surgeon at the end of the operation and met the inclusion criteria (Fig. 1). The majority of patients were male (63%), and the median age was 70 years [interquartile range: 62–77]. In 265 patients (9.5%), the tumor was not completely removed. Patient and tumor characteristics according to resection status are shown in Table 1.

**Table 1:** Patient and tumor characteristics

Patient characteristics	Total		Complete tumor removal		Incomplete tumor removal		*P* value
	*n* = 2799		*n* = 2534 (90.5%)		*n* = 265 (9.5%)		
	*n*	%	*n*	%	*n*	%	
*Gender*							0.044
Man	1754	63	1603	63	151	57	
Women	1045	37	931	37	114	43	
*Age (in groups)*							0.142
< 65 years	888	32	791	31	97	37	
65–74 years	959	34	880	35	79	30	
> 75 years	952	34	863	34	89	34	
*Charlson score*							0.119
0	1243	44	1121	44	122	46	
1	634	23	587	23	47	18	
2+	922	33	826	33	96	36	
*ASA score*							0.17
I–II	1942	70	1768	70	174	66	
III+	838	30	749	30	89	34	
Unknown							
*Location of tumor*							< 0.001
Esophageal-gastric junction/fundus	313	12	286	12	27	11	
Corpus	833	31	775	31	58	23	
Antrum/pylorus	1330	49	1214	49	116	45	
Entire stomach	141	5	94	4	47	18	
Pouch/anastomosis	109	4	99	4	10	4	
Unknown	0	0	0	0	0	0	
*Clinical tumor category*							< 0.001
cT0-2	825	30	781	31	44	17	
cT3	1177	43	1041	42	136	52	
cT4	152	6	128	5	24	9	
cTx	602	22	542	22	60	23	
Missing	0	0	0	0	0	0	
*Clinical node category*							0.007
cN0	1433	52	1319	53	114	43	
cN+	1038	38	925	37	113	43	
cNx	288	10	251	10	37	14	
Missing	0	0	0	0	0	0	
*Clinical metastases category*							0.043
cM-0	2618	94	2375	94	243	92	
cM+	44	2	35	1	9	3	
cMx	137	5	124	5	13	5	
*Tumor histology*							0.092
Adenocarcinoma	2633	95	2379	95	254	97	
Squamous carcinoma	5	0	5	0	0	0	
Other	133	5	127	5	6	2	
Not applicable	3	0	2	0	1	0	
Unknown	0	0	0	0	0	0	
*Histological subtype*							< 0.001
Intestinal adenocarcinoma	1149	41	1097	43	52	20	
Diffuse adenocarcinoma	823	29	676	27	147	56	
Mixed type	164	6	153	6	11	4	
Unknown	663	24	608	24	55	21	
*Differentiation grade*							< 0.001
Well/moderate	881	32	835	33	46	17	
Poor/undifferentiated	1471	53	1273	50	198	75	
Not available	93	3	91	4	2	1	
Unknown	354	13	335	13	19	7	
*Pathological tumor stage*							< 0.001
pT0-2	1030	37	1010	40	20	8	
pT3	1094	40	977	39	117	45	
pT4	614	22	490	20	124	47	
pTx	32	1	30	1	2	1	
Missing	0	0	0	0	0	0	
*Pathological node stage*							< 0.001
pN0	1254	45	1198	48	56	21	
pN+	1481	53	1277	51	204	78	
pNx	36	1	33	1	3	1	
Missing	0	0	0	0	0	0	
*Pathological metastases stage*							< 0.001
pM0	2490	89	2272	90	218	82	
pM1	118	4	89	4	29	11	
pMx	54	2	47	2	7	3	
Not applicable	137	5	126	5	11	4	
*cT versus pT staging*							0.019
Correct estimated	549	20	509	20	40	15	
Underestimated T stage	256	9	218	9	38	14	
Overestimated T stage	72	3	68	3	4	2	
cTx	283	10	256	10	27	10	
pTx	21	1	20	1	1	0	
cT or pT missing	0	0	0	0	0	0	
Not applicable (neoadjuvant therapy)	1585	57	1431	57	154	58	

*ASA* American Society of Anesthesiologists

Tumor location, histological subtype, and differentiation grade were statistically significant different between patient with complete or incomplete tumor removal. Clinical and pathological *T*-, *N*- and *M*-stage was more advanced in patients with incomplete tumor removal.

### Risk Factors for Incomplete Tumor Removal

A tumor located in the entire stomach (versus gastroesophageal junction/fundus), higher cT-categories (cT3, cT4 and cTx category versus cT0-2), a pN+-category and pNx-category (versus pN−), and diffuse or unknown type adenocarcinoma (versus intestinal type) were associated with incomplete tumor removal (Table 2). Unknown differentiation grade was associated with a complete tumor removal (compared with poor differentiation grade/undifferentiated). The area under the ROC of the multivariate model was 0.76.

**Table 2:** Probability for incomplete tumor removal, results of uni- and multi-variable analyses

Probability for incomplete tumor removal	Univariable analysis			Multivariable analysis		
Variables	*n*	OR [95% CI]	*P* value	*n*	OR [95% CI]	*P* value
**Total**	**2799**			**2671**		
*Age (year)*			0.143			
0–64	888	1				
65–74	959	0.73 [0.54–1]	0.050			
75+	952	0.84 [0.62–1.14]	0.263			
*Charlson score*			0.121			
0	1243	1				
1	634	0.74 [0.52–1.05]	0.087			
2+	922	1.07 [0.81–1.42]	0.648			
*ASA score*			0.170			
I/II	1942	1				
III+	838	1.21 [0.92–1.58]				
*Tumor location*			< 0.001			< 0.001
GEJ/Fundus	313	1		304	1	
Corpus	833	0.79 [0.49–1.28]	0.339	813	0.70 [0.43–1.16]	0.167
Antrum/pylorus	1330	1.01 [0.65–1.57]	0.957	1308	0.95 [0.60–1.50]	0.828
Entire stomach	141	5.30 [3.13–8.98]	< 0.001	138	3.38 [1.91–5.96]	< 0.001
Pouch/residual stomach	109	5.30 [3.13–8.98]	0.862	108	1.14 [0.51–2.57]	0.749
*Clinical tumor category*			< 0.001			0.005
cT0-2	825	1		808	1	
cT3	1177	2.32 [1.63–3.30]	< 0.001	1137	1.75 [1.20–2.56]	0.004
cT4	152	3.33 [1.96–5.66]	< 0.001	146	2.63 [1.47–4.70]	0.001
cTx	602	1.97 [1.31–2.94]	0.001	580	1.60 [1.03–2.48]	0.036
*Pathological node category*			< 0.001			< 0.001
pN−	1254	1		1207	1	
pN+	1481	3.42 [2.52–4.64]	< 0.001	1433	2.73 [1.96–3.80]	< 0.001
pNx	36	1.95 [0.58–6.53]	0.282	31	3.17 [0.86–11.61]	0.082
*Clinical metastases category*			0.053			0.984
cM0	2618	1		2517	1	
cM1	44	2.51 [1.19–5.29]	0.015	43	1.08 [0.46–2.51]	0.867
cMx	137	1.03 [0.57–1.84]	0.935	111	1.02 [0.53–1.99]	0.948
*Histological subtype*			< 0.001			< 0.001
Intestinal adenocarcinoma	1149	1		1112	1	
Diffuse adenocarcinoma	823	4.59 [3.30–6.38]	< 0.001	797	3.15 [2.14–4.64]	< 0.001
Mixed type	164	1.52 [0.77–2.97]	0.224	160	1.02 [0.50–2.06]	0.963
Unknown	663	1.91 [1.29–2.82]	0.001	602	2.05 [1.34–3.13]	0.001
*Differentiation grade*			< 0.001			0.017
Well/moderate	881	0.35 [0.25–0.49]		839	0.72 [0.48–1.06]	
Poor/undifferentiated	1471	1	< 0.001	1417	1	0.096
Not applicable	93	0.14 [0.04–0.58]	0.006	89	0.33 [0.08–1.38]	0.129
Unknown	354	0.37 [0.22–0.59]	< 0.001	326	0.50 [0.30–0.83]	0.008
*Year of resection*			0.562			
2011	250	1				
2012	319	0.71 [0.42–1.20]	0.199			
2013	448	0.71 [0.43–1.15]	0.160			
2014	498	0.78 [0.49–1.25]	0.294			
2015	419	0.66 [0.40–1.09]	0.104			
2016	475	0.64 [0.39–1.05]	0.078			
2017	390	0.61 [0.36–1.02]	0.061			

*ASA* American Society of Anesthesiologists

### Hospital Volume

**Fig. 2:**
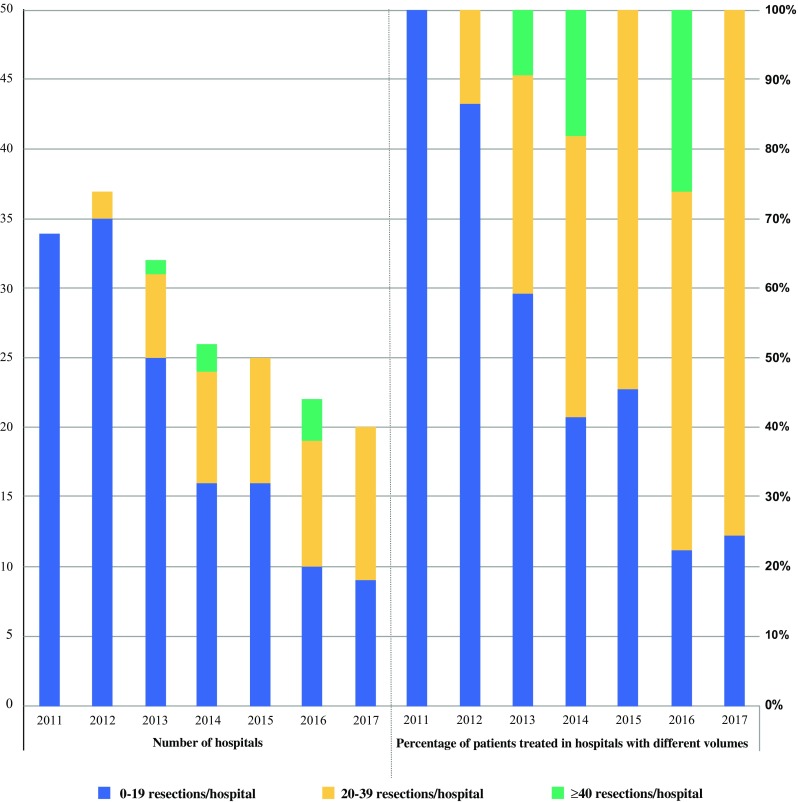
Centralization gastric cancer surgery

In Fig. 2, the centralization of gastric surgery in the Netherlands is shown. Compared with 2011, the hospital volumes were higher in 2017, and the number of hospitals performing gastric surgery decreased.

In all logistic regression models, annual hospital volume of < 20 was associated with a higher percentage of incomplete tumor removal compared to annual hospital volumes of 20–39 and ≥ 40 resections per year (Table 3). There was no statistically significant difference between 20–39 and ≥ 40 resections per year. In a sensitivity analysis including data from the period 2014–2017, similar results were found (data not shown). In stratified analyses according to neo-adjuvant therapy, similar results were found. Patients not treated with neo-adjuvant therapy, with a volume of < 20 resections/year had a higher probability for incomplete tumor resection compared to 20–39 resections/year and 40 or more resections/year (OR: 0.60 [0.37–0.98] and 0.19 [0.05–0.69], respectively). In patients treated with neo-adjuvant therapy, the probability for incomplete tumor resection also was higher for low hospital volume (< 20 resections/year) compared with 20–39 resections/year (OR: 0.65 [0.43–0.98]) and for 40 or more resections/year (OR 0.50 [0.23–1.09]).

**Table 3:** Multiple regression models to test the association of hospital volume with incomplete tumor removal

Probability for incomplete tumor removal based on patient and tumor characteristics 2011–2017		*n*	OR	95% CI	*P* value	Nagelkerke *R*^2^	ROC
	**Total**	2671				0.17	0.76
Patient and tumor factors added to the model: location tumor, cT category, pN stage, cM category, histological subtype, differentiation grade							
*Hospital volume*					0.001		
Not adjusted	< 20 resections/year	1388	1				
	20–39 resections/year	1155	0.68	[0.52–0.89]	0.004		
	40 or more resections/year	256	0.41	[0.23–0.74]	0.003		
*Hospital volume*					< 0.001		
Adjusted for: location tumor, cT category, pN stage, cM category, histological subtype, differentiation grade	< 20 resections/year	1308	1				
	20–39 resections/year	1134	0.56	[0.42–0.76]	< 0.001		
	40 or more resections/year	229	0.34	[0.18–0.64]	0.001		
*Hospital volume (other reference)*					< 0.001		
Adjusted for: location tumor, cT category, pN stage, cM category, histological subtype, differentiation grade	< 20 resections/year	1308	2.95	[1.57–5.55]	0.001		
	20–39 resections/year	1134	1.66	[0.88–3.13]	0.120		
	40 or more resections/year	229	1				

### Site of Tumor-Positive Margin

In 175 of 265 patients with incomplete tumor removal, the site of the tumor-positive resection margin was reported in the DUCA (Supplementary Table 1). When the resection of the tumor was incomplete, the proximal resection margin was mostly involved in patients with proximal gastric cancer (junctional/fundus 86% and corpus 80%). Gastrectomy for distal tumors (antrum/pylorus) was most often incomplete at the distal margin (68%). When the tumor was located in the entire stomach, the resection was incomplete at the distal margin in 17%, the proximal margin in 42%, and involvement of both margins was seen in 42% of patients (Supplementary Table 2).

## Discussion

This Dutch cohort study shows that patients with advanced gastric cancers (i.e., involving the entire stomach, advanced TNM-stage, and diffuse-type gastric cancer) are at risk for incomplete tumor removal. Furthermore, low annual hospital volume (< 20 resections per year) also is associated with a higher risk for incomplete tumor removal than middle and high-volume hospitals. The present study is the first population-based study reporting patient-related and tumor-related factors associated with incomplete tumor removal for gastric cancer. The risk factors that were identified in this national cohort study are similar to earlier studies: Songun et al.^17^ reported the association between incomplete tumor removal with tumor location and size of the tumor. Other studies reported the association between incomplete tumor removal and diffuse type carcinoma.^18^,^19^ The risk factors identified in the present study appear to be related to more advanced stomach cancer, and this in itself might be a risk factor for an incomplete tumor removal.

In addition to patient and tumor factors, Bissolati et al. studied the association between the distance from the tumor to the margin of resection and incomplete tumor removal. They showed that resection margins of < 20 mm in T1 tumors resection and resection margins of < 30 mm in and T2-4 tumors were associated with incomplete tumor removal.^7^ In the present study, the association of resection margin with incomplete tumor removal could not be assessed. Based on the study by Bissolati et al., it could be argued that an extra wide resection margin may prevent incomplete tumor removal. The Dutch guideline recommends a minimum resection margin of 60 mm.^6^ The German guideline recommends a resection margin of 50 mm for intestinal type and 80 mm for diffuse-type gastric cancer.^20^

Choosing an appropriate surgical margin can be challenging. The margin should be wide enough to prevent incomplete tumor removal but at the same time a technically feasible and reliable reconstruction should be created. To achieve a safe proximal resection margin for middle gastric tumors, a total gastrectomy may be indicated. Although postoperative mortality and 5-year survival after total and subtotal gastrectomy is comparably, a subtotal gastrectomy is associated with less nutritional side effects and a better quality of life.^21^

For proximal gastric tumors that invade the esophagus, a more technically challenging anastomosis in the lower mediastinum or a total gastrectomy with subtotal esophagectomy and colonic interposition may be indicated. This procedures have a higher risk for anastomotic leakage or other postoperative complications.^22^,^23^

Bissolati et al. also showed that there was an association between incomplete tumor removal at the esophagogastric junction. However, the surgeon may be confronted intraoperatively with a difficult decision as how to deal with suspicious extension of the tumor beyond what was anticipated. Proximal gastric cancers may invade the esophagus and the proximal resection margin is at risk.

In the present study, tumor location was not associated with incomplete tumor removal. Distal gastric cancers may invade the duodenum and a Whipple’s operation for patients who can tolerate this should be considered. In the Netherlands, the foundation for oncological cooperation (SONCOS) recommends that gastric and esophageal resections should be performed in the same hospital.^24^ However, there are no recommendations regarding the combination of gastric and hepatobiliary surgery.^25^ Therefore, when it is anticipated that the proximal margin at the esophagus or the duodenum is at risk, it is probably advisable to refer patients to hospitals where esophageal and/or hepatobiliary surgery is performed.

To facilitate a radical resection without unnecessarily wide resection margins, intraoperative frozen-section analysis could be used. However, this technique is time-consuming, and the clinical value can be dubious since results can be false negative.^26^,^27^ Squires et al. evaluated outcomes of patients with gastric cancer with a positive intraoperative proximal frozen section converted to an R0 resection in the same procedure. The local recurrence was significantly lower in the converted-to-R0 group than in patients with a positive final frozen section. This study showed that overall survival and progression-free survival was not improved.^28^ If time is a concern of hospitals, a frozen section could be considered to achieve a *R*0 resection in high-risk patients as identified in this study rather than in all patients.

Additionally, intraoperative endoscopic ultrasonography may help to determine the extent of infiltration in the esophagus or duodenum.^29^ Kawakatsu et al. described the combination of preoperative placement of marking clips and intraoperative endoscopy as being helpful to determine a surgical margin in patients who undergo laparoscopic gastrectomy. However, this is the only study that describes the systematical use of endoscopy during gastrectomy. Further studies are needed to evaluate the benefits of this technique.

Besides tumor-related factors, the surgeon’s experience with esophageal and gastric cancer surgery and the number of operations per year performed (hospital volume) may be important to reduce the number of incomplete resections. In the present study, a hospital volume of < 20 gastric resections per year was associated with a higher chance of incomplete tumor removal compared with 20–50 and > 50 resections per year. In the past, the association between hospital volume and postoperative morbidity/mortality and overall survival has been studied.^8^–^10^,^30^–^33^ For overall survival, conflicting results were published. However, for postoperative morbidity and mortality, several studies reported improved outcomes in high-volume centers. More recently, low hospital volume (< 25 resections per year) was associated with fewer retrieved lymph nodes.^34^ Between 2012 and 2014, the Association of Surgeons of the Netherlands introduced volume standards for complex surgery. In particular for gastric surgery, a minimum volume of 10 gastric cancer resections in 2012, and from 2013 onwards a minimum of 20 resections per year was required. Currently, some Dutch hospitals have not met this standard yet, and centralization in gastric surgery is still ongoing (Fig. 2). It may be possible that hospitals with a relatively low number of patients with gastric cancer use more liberal criteria to select patients for gastrectomy to comply with the minimum required target. This may result in worse outcomes, e.g., higher rates of incomplete tumor removal. At present, we are performing a more in-depth examination in several hospitals to identify if organizational, human, or technical factors contribute to unfavorable outcomes after gastrectomy. Nevertheless, the current study endorses the need for centralization of gastric cancer surgery. Another strategy could involve discussing complex patients in a multicenter, multidisciplinary team.^35^

In the case of postoperative determination of tumor-positive resection margins, some studies describe that adjuvant chemoradiotherapy is associated with improved survival, especially for patients who had no neoadjuvant therapy.^36^–^38^ Another option that may benefit is to perform a reoperation with resection of the tumor-positive resection margins.^39^,^40^ The largest cohort of reoperations was 122 patients, and a reoperation was successfully performed in 41% of these patients. The authors of the study describe a survival benefit especially for stage N2 or lower tumors.^41^ However, evidence for an optimal treatment after an incomplete tumor removal is based on nonrandomized studies with small patient groups.

The main strength of this study is the nationwide coverage of the dataset allowing national performance to be assessed. Outcomes of studies using population-based data reflect daily clinical practice. Prospective (randomized) trials are usually conducted under strict quality control and only with selected patients and thus may not reflect the real world. A national registry might do reflect the real world. However, a database from a national registry also may have its disadvantages; the accuracy and completeness of data may be questioned. Nevertheless, we believe that the DUCA database is accurate to answer our research question. The case ascertainment of the DUCA database is estimated at 97.8%, and the resection status is reported with high completeness (1.6% missing; Fig. 1).^13^ Because in the Netherlands, the information regarding resection margins must be reported according to a standardized pathology report. We assume that the accuracy of the registered resection status also is high.^12^

Another limitation is the retrospective nature of this study. In this study, we could not evaluate the influence of treatment-related factors on resection status, such as neo-adjuvant chemotherapy and the surgical approach. The reason for this is that bias in the selection of patients for specific treatments may have occurred (treatment by indication bias). Therefore, this study could not evaluate much-discussed, treatment-related factors, such as the approach of surgery. However, recently, a study with data of the DUCA compared minimally invasive gastrectomy with open gastrectomy in a propensity-matched cohort. This study showed no differences in resection status between the two groups (*R*0 in 88% vs. 85%, *P* = 0.189).^42^ Another potential treatment-related factor that could not be evaluated in this study is inadequate diagnostic staging. From the present dataset, it was not possible to compare the diagnostic workup between patients who underwent a complete and incomplete tumor removal, because both patient identity and hospital identity are anonymous.

Finally, data on survival were not available. Therefore, evaluation of the (independent) association of complete resection with survival was not possible. A gastrectomy with tumor-positive margins may reflect an aggressive biology of the tumor and as a consequence have a poor prognosis. Even after gastrectomy with negative resection margins, large, poorly differentiated tumors will likely spread beyond the surgical field, and surgery cannot cure these patients. Future studies may be needed to evaluate the independent association of incomplete resections with survival.

## Electronic supplementary material

Below is the link to the electronic supplementary material.

Supplementary material 1 (DOCX 12 kb)
