# Supplementary material for: Population-Based Study on Risk Factors for Tumor-Positive Resection Margins in Patients with Gastric Cancer
Source: Ann Surg Oncol. 2019 Apr 22;26(7):2222–33. doi: 10.1245/s10434-019-07381-0 (PMC6545177; doi:10.1245/s10434-019-07381-0)
Supplement: Supplementary file 1 — Supplementary material 1 (DOCX 12 kb) [file 10434_2019_7381_MOESM1_ESM.docx]

**Supplementary Table 1** Location tumor-positive resection margin

| **Location tumor-positive resection margin** |  |  |
| --- | --- | --- |
|  | n | % |
| Distal | 68 | 26% |
| Proximal | 78 | 29% |
| Circumferential | 1 | 0.4% |
| Distal and proximal | 29 | 11% |
| Unknown | 90 | 34% |
| *Reported unknown* | *37* | *41%* |
| *Both margins reported >0 mm* | *43* | *48%* |
| *Missing* | *6* | *6.7%* |
| *Reported not applicable* | *3* | *0.3%* |
| Total | 265 |  |

**Supplementary Table 2** Location tumor-positive resection margin for each tumor location

|  |  |  | **Location tumor-positive resection margin** | | | | | |  |  |
| --- | --- | --- | --- | --- | --- | --- | --- | --- | --- | --- |
|  | *Total* | No information | **Distal** | | **Proximal** | | **Distal and proximal** | | **Circumferential** | |
|  | n | n | n | % | n | % | n | % |  |  |
| **Location of tumor** |  |  |  |  |  |  |  |  |  |  |
| GEJ | 14 | *6* | 0 | 0% | 7 | 88% | 0 | 0% | 1 | 13% |
| Fundus | 13 | *6* | 1 | 14% | 5 | 71% | 1 | 14% |  |  |
| Corpus | 58 | *18* | 6 | 15% | 32 | 80% | 2 | 5% |  |  |
| Antrum/pylorus | 116 | *38* | 53 | 68% | 16 | 21% | 9 | 12% |  |  |
| Entire stomach | 47 | *11* | 6 | 17% | 15 | 42% | 15 | 42% |  |  |
| Pouch/anastomosis | 10 | *5* | 2 | 40% | 2 | 40% | 1 | 20% |  |  |

*GEJ* gastroesophageal junction
